# Modulation of Alpha Oscillations in the Human EEG with Facial Preference

**DOI:** 10.1371/journal.pone.0138153

**Published:** 2015-09-22

**Authors:** Jae-Hwan Kang, Su Jin Kim, Yang Seok Cho, Sung-Phil Kim

**Affiliations:** 1 Department of Brain and Cognitive Engineering, Korea University, Seoul, Republic of Korea; 2 Department of Psychology, Korea University, Seoul, Republic of Korea; 3 Department of Human and Systems Engineering, Ulsan National Institute of Science and Technology, Ulsan, Republic of Korea; University of Montreal, CANADA

## Abstract

Facial preference that results from the processing of facial information plays an important role in social interactions as well as the selection of a mate, friend, candidate, or favorite actor. However, it still remains elusive which brain regions are implicated in the neural mechanisms underlying facial preference, and how neural activities in these regions are modulated during the formation of facial preference. In the present study, we investigated the modulation of electroencephalography (EEG) oscillatory power with facial preference. For the reliable assessments of facial preference, we designed a series of passive viewing and active choice tasks. In the former task, twenty-four face stimuli were passively viewed by participants for multiple times in random order. In the latter task, the same stimuli were then evaluated by participants for their facial preference judgments. In both tasks, significant differences between the preferred and non-preferred faces groups were found in alpha band power (8–13 Hz) but not in other frequency bands. The preferred faces generated more decreases in alpha power. During the passive viewing task, significant differences in alpha power between the preferred and non-preferred face groups were observed at the left frontal regions in the early (0.15–0.4 s) period during the 1-s presentation. By contrast, during the active choice task when participants consecutively watched the first and second face for 1 s and then selected the preferred one, an alpha power difference was found for the late (0.65–0.8 s) period over the whole brain during the first face presentation and over the posterior regions during the second face presentation. These results demonstrate that the modulation of alpha activity by facial preference is a top-down process, which requires additional cognitive resources to facilitate information processing of the preferred faces that capture more visual attention than the non-preferred faces.

## Introduction

An individual human face contains a wealth of information that conveys unique characteristics of identity, gender, age, and emotional state to others. Featural and configural information in faces constructs facial identity and attractiveness and gives rise to specific emotions and facial preference in others [1–4]. Outcomes from relevant facial information processing not only highly affect the selection of a mate or friends [5] but can also determine the winner of a political election [6–8]. For these reasons, numerous studies to understand facial information have been conducted across the evolutionary, psychological, neuroscience, and neuromarketing fields for decades [1,7,9].

In this regard, neural activities recorded by a variety of electrophysiological or neuroimaging techniques, such as functional magnetic resonance imaging (fMRI), functional near-infrared spectroscopy (fNIRS), magnetoencephalography (MEG), and electroencephalography (EEG) have provided important clues to understand a series of face-specific cognitive processes as well as their neural substrates. Based on the considerable evidence revealed by these techniques, it has been well documented that facial information processing involves specific spatiotemporal characteristics of neural activities in the brain. From the perspective of spatial characteristics, numerous neuroimaging studies have demonstrated that facial information processing is based on relevant activities in face-specific cortical and subcortical structures such as the fusiform face area, occipital face area, superior temporal sulcus (STS), bilateral medial STS, bilateral amygdala, bilateral inferior frontal gyrus, precuneus, and anterior paracingulate cortex [10,11]. From the perspective of temporal characteristics, EEG or MEG studies measuring event-related potentials (ERPs) and oscillations (EROs) have shown that facial information processing engages both bottom-up and top-down processes that are temporally composed of facial perception [12,13], recognition [14], identification [15,16], familiarity [17], attractiveness [18,19], and preference [20,21].

Certain processes related to automatic and instant responses to facial presentation, such as perception, recognition, and identification, have a tendency to modulate the early components of ERPs such as P1 [22], N170 [23,24], and high frequency oscillations such as local gamma activity [2,25,26]. In contrast, processing of facial beauty and preference is related to the late components of ERPs such as EPN (early posterior negativity, ~250 ms), LPC (late parietal component, 400–600 ms), P300 and LPP (late positive potential) [15,19,27], and slow frequency oscillations such as alpha activity (8–13 Hz) across global brain regions [28,29].

Among the several processes for facial information described above, in the present study, we focused on the investigation of subjective facial preference. Subjective facial preference is gradually formed throughout facial information processing and firmly established at the last stage just before the participant’s actions such as specific selections or decision-making. Therefore, it is important to understand the course of the formation of facial processing in the brain in order to identify neural correlates of facial preference and to predict humans’ attitudes or responses to a specific face in individuals or a group. Several studies have reported such neural correlates of facial preference. An fMRI study reported that the cognitive process for facial preference was implicated in reward-related brain structures such as the nucleus accumbens and orbitofrontal cortex [30] but did not report any temporal properties of neural processing of facial information. Lindsen *et al*. analyzed EEG signals recorded during a preferential decision task between two sequentially presented face stimuli and reported sustained posterior positive ERPs during the formation of positive impression of the first face and modulation of frontal theta and posterior gamma power related to the formation of a preference. They allowed participants unrestricted viewing times of face stimuli for ensuring proper judgment. However, this resulted in large variations in response time and permitted ambiguity in the interpretation of neural correlates due to time-unlocking EEG activities [20]. A recent EEG study on facial preference reported successful classification between preferred and non-preferred faces using an artificial neural network with 0.743 and 0.914 accuracy rates at participant independent and dependent levels, respectively [21]. However, this study did not focus on either the cognitive processes or the spatiotemporal characteristics of EEG oscillations underlying the formation of facial information processing.

In the present study, we aimed to investigate how brain activity represented in the form of EEG oscillations varied during the course of facial information processing. We designed two independent facial preference tasks, including a passive viewing task and an active choice task, to rule out a possible overlap between facial preference and other processes and to generate time-locked EEG signals modulated by facial preference. We expected that spectral analyses of the EEG signals recorded during both tasks would endow us with neurophysiological evidence of the formation of facial preference by means of changes in EEG oscillatory rhythms. Based on the properties of facial preference that are strongly tied to emotional and top-down processes, we hypothesized that facial preference would modulate low-frequency oscillatory rhythms more than high-frequency rhythms and be localized over the posterior region, which has been known as a key region for manipulating emotional and social interaction processes [28,31–34]. In addition, we hypothesized that spatiotemporal characteristics of EEG oscillatory rhythms during the formation of facial preference would be dissimilar between the passive viewing and active choice tasks. Such task-specific patterns of oscillatory power could shed some light on the neural processing for facial preference formation.

## Materials and Methods

### Experiment

#### Participants

The research protocol was reviewed and approved by the Korea University Institutional Review Board (IRB) (IRB Number: KU-IRB-13-28-A-1). The study was conducted in accordance with the Declaration of Helsinki. Informed consent was obtained in writing from each participant following the protocol approved by the IRB before the study began. Sixteen university students (8 male) with an age range between 22 and 28 years old participated in this study and were paid 15,000 KRW for their participation. All participants were right-handed and had normal or corrected-to-normal vision.

#### Stimuli

The stimuli consisted of 36 color photos of frontal faces with neutral expressions taken from 18 males and 18 females (all native Koreans). All images were equalized in brightness and contrast, adjusted into 113 × 151 pixels, and matched in height to the middle of the forehead and nose. The background color of the images was set to a light gray, but the colors of the hair and clothes were not modified. All the image processing procedures were performed using Adobe Photoshop (CS5, Adobe). The face stimuli were then divided into a set of 24 experimental stimuli (12 male) used for the analysis and another set of 12 filler stimuli (6 male) used only for checking participants’ consistent engagement in the experiment. Unlike the experimental stimuli, the filler stimuli were marked with a red dot on the nose, and the EEG signals recorded during the presentation of the filler stimuli were excluded from the analysis. The stimuli were displayed on a 22-inch monitor (1920 × 1080 pixels) at a distance of approximately 70 cm from the participant.

#### Tasks

**Fig 1:**
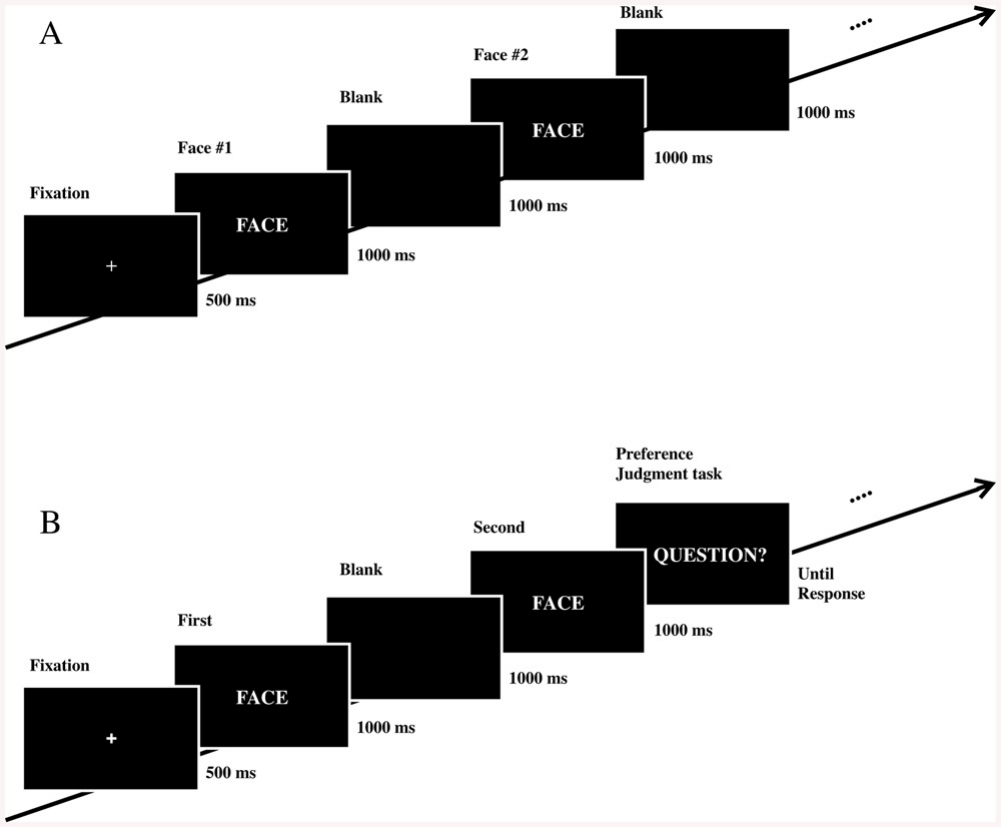
Experimental paradigms of the passive viewing task (A) and the active choice task (B).

Each participant sat on a chair and performed two different experimental tasks in the electrically shielded darkroom, including a passive viewing task (Fig 1A) and an active choice task (Fig 1B). The 24 experimental stimuli were used in both the passive viewing and active choice tasks, whereas the 12 filler stimuli were used only for the passive viewing task.

In the passive viewing task, after a 500-ms initial fixation interval (a cross appeared during this interval), one of the 36 face stimuli was presented for 1 s followed by a 1-s inter-stimulus interval (ISI) with a blank screen. When an experimental stimulus was presented, participants were asked to passively watch the image with no explicit response. When a filler stimulus was presented, the participants were asked to make a preference decision by pressing the “f” key for an unattractive face or the “j” key for an attractive face during the preference judgment period after a 1-s ISI. The next trial did not begin until the judgment was completed. The order of stimulus presentation was determined pseudo-randomly. Each of the 24 experimental stimuli was presented six times, resulting in a total of 144 trials. In addition, each filler stimulus was presented six times.

In the active choice task, designed to assess the preference value of each face stimulus, each trial began with an initial fixation period (0.5 s), and a pair of same-sex face stimuli were presented sequentially, each for 1 s with a 1-s ISI (Fig 1B). After the presentation of the two consecutive stimuli, participants were shown a written question, “Which face do you prefer, the former or latter?” on the screen and answered it by pressing the “f” key for the former or the “j” key for the latter. The next trial did not begin until participants made their response. All possible pairs among the 12 face stimuli for each sex were created (i.e., a total of _12_C_11_ = 66 pairs per sex). Each pair was presented twice by switching the order of stimuli within the pair. Consequently, the active choice task consisted of a total of 264 trials (i.e., a total of _12_P_11_ = 132 trials per sex). The order of presentation of each ordered pair was randomized.

While participants performed both tasks, EEG signals were simultaneously recorded using the ActiveTwo amplifier (BioSemi, Amsterdam, The Netherlands) with a sampling rate of 1 KHz from 60 Ag/AgCl scalp electrodes attached to the scalp according to the extended 10–20 international system. All electrode impedances were kept below 5 KΩ. The ground electrode was positioned on the forehead, and the left and right linked mastoids were used as reference. The EEG signals were filtered on-line between 0.01 and 100 Hz. The vertical and horizontal EOG signals were recorded from electrodes placed 1.5 cm lateral to the left and right external canthi to monitor eye movement and blinks.

### Face preference analysis

We quantified the preference of each face stimulus and examined the effects of gender and presentation order on preference judgments. First, we defined a face preference value (FPV) of a stimulus as a ratio of the number of trials the stimulus was chosen to the total number trials it was presented during the active choice task. We calculated the FPV per participant by examining how many times each face stimulus was chosen as a preferred face by a participant (S1 File). For instance, if a face stimulus was chosen by a participant 10 times, the FPV was 0.45 (10/22 because a stimulus was always presented 22 times to each participant). The FPV varied between 0 and 1 (0 = never chosen, 1 = always chosen). Second, we divided the overall FPVs into same-sex and opposite-sex subsets and examined gender differences in FPVs between same-sex and opposite-sex conditions. The same-sex subset contained the FPVs in the case when the face pair shown to a participant was of the same sex and the opposite-sex subset contained the FPVs when the face pair shown to a participant was of the opposite sex. A paired *t*-test analysis was repetitively used for each face to examine the effect of gender on FPVs. Third, we examined whether the order of stimulus presentation influenced the participants’ selection by performing a paired *t*-test (first versus second order) in all participants. Finally, every face stimulus was sorted in descending order based on its average FPV, creating a ranking of the face stimuli. Then, the ranked stimuli were divided into three groups (preferred, upper 33%; neutral, mid 33%; non-preferred, lower 33%). We examined whether there was a difference in FPVs across these groups by conducting a one-way repeated measures ANOVA with the factor of preference (3: preferred/neutral/non-preferred) and a post-hoc *t*-test analysis.

### EEG analysis

#### EEG preprocessing

All recorded EEG signals during both the passive viewing and active choice tasks were analyzed off-line as follows: The EEG data were re-sampled to a 500-Hz sampling rate, band-pass filtered between 0.5 and 55 Hz, and re-referenced by means of the common average reference (CAR) method [35,36]. To eliminate EOG and EMG noises, we performed an independent component analysis (ICA) on the EEG data using the EEGLAB toolbox [37]. The independent components in association with eye blinks or movements were identified and eliminated by visual inspection. The corrected independent components were used to reconstruct noise-reduced EEG data. Then, the EEG data in both tasks were segmented with an epoch of 1.5 s, extending from 0.5 s before stimulus onset to 1.0 s post-stimulus, and appended by some residual samples to avoid edge artifacts that might be caused by data filtering or power calculation. For the subsequent analyses, the EEG data were grouped into three sets of epochs: The first set consisted of 144 epochs in the passive viewing task, the second set consisted of 264 EEG epochs for the stimuli presented first in the active choice task, and the third set consisted of 264 EEG epochs for the second stimuli in the active choice task.

#### EEG oscillatory power calculation

To calculate EEG oscillatory power, we employed a complex wavelet transform based on the procedure suggested by Tallon-Baudry *et al*. [38]. The epoched EEG data were convolved with a complex Morlet wavelet: ${\text{w(t,f}}_{\text{0}}\text{) = A}\cdot {\text{e}}^{\text{-(}{\text{-t}}^{\text{2}}/{\text{2}\sigma }_{\text{t}}^{\text{2}}\text{)}}\cdot {\text{e}}^{{\text{2i}\pi \text{f}}_{\text{0}}\text{t}}$ with a normalization factor $\text{A=}\sqrt{\text{1}/{\text{(}\sigma }_{\text{t}}\sqrt{\pi }\text{)}}$. Here, we set f_0_/σ_f_ = 5 where σ_f_ = 1/(2πσ_t_). f_0_ denotes the central frequency and σ_t_,σ_f_ are the standard deviation of the waveform in time and frequency, respectively [38]. This procedure was applied to individual central frequencies ranging from 2 to 55 Hz in steps of 0.5 Hz. Oscillatory power was calculated by the absolute value of the complex value of the wavelet-transformed signals. All the power values were log-transformed and normalized by subtracting the mean value of the baseline period for each frequency, where the baseline period was determined as the 200 ms before the onset of the first stimulus presentation in each epoch.

#### Statistical analysis of EEG data

We investigated the modulation of EEG oscillatory power by face stimuli with different FPVs. We performed statistical analyses of each of the three EEG data sets created as described above. Two statistical analyses were used to investigate: 1) differences in the power of EEG oscillations within a specific frequency band in response to face stimuli with different FPVs and 2) correlations between the power of EEG oscillations and the FPV.

For the statistical analysis, EEG power data were refined in terms of frequency band and post-stimulus time period. For frequency, EEG oscillatory powers were segmented into five bands such as theta (4–8 Hz), alpha (8–13 Hz), low beta (13–20 Hz), high beta (20–30 Hz), and gamma (30–55 Hz) bands. The power values within each band were averaged over frequency. For time periods, the EEG power data were segmented into 10 post-stimulus time periods each of which spanned 0.1 s without overlap. In so doing, we obtained 50 points of analysis (5 frequency bands × 10 time segments) for each EEG channel. Then, we performed the statistical analyses on each point of analysis. First, repeated measures ANOVA and post-hoc *t*-test analyses were used to assess the statistical differences in oscillatory power between two (preferred and non-preferred) or three (preferred, neutral, and non-preferred) preference groups. Second, linear correlation analyses examined correlations between oscillatory powers and FPV. All the *p*-values in these analyses were corrected by the false-discovery rate (FDR) for multiple comparisons. Lastly, we represented the statistical results in a spatial map across the brain. For this topographical representation, 60 EEG sites were grouped into 10 disjoint brain areas (prefrontal: FP1/z/2, AF3/4; left frontal: F3/5/7, FT3/5/7; mid frontal: F1/z/2, FC1/z/2; right frontal: F4/6/8, FC4/6/8; left central: C3/5, CP3/5, T6, TP7; mid central: C1/z/2, CP1/z/2; right central: C4/6, CP4/6, T8, TP8; left occipital: P1/3/5/7, PO3/5/7,O1; mid occipital: P1/z/2, POz, Oz; right occipital: P4/6/8, PO4/6/8, O2).

## Results

### Behavior results

Prior to the analysis of EEG oscillatory power relative to FPVs, we investigated the gender effect on preference judgment. Paired *t*-tests examined statistical differences in FPVs of each stimulus and revealed that no face stimulus used in this study was biased by the participants’ gender [*ps* > 0.01]. Next, we examined whether the participants selected the first or second face stimulus as a preferred face according to its presentation order. Across all the participants, the overall percentage of the first and second stimulus selection was 0.49±0.017 and 0.51±0.017, respectively. A paired *t*-test revealed no significant difference [*t*(15) = 1.45, *p* = 0.168], indicating that the participants did not select the preferred face by means of the order of stimulus presentation. Finally, we divided all the face stimuli into three different groups according to the ranking of FPVs. The means and standard deviations of FPVs in each group were 0.80±0.06 for the preferred, 0.49±0.13 for the neutral, and 0.22±0.11 for the non-preferred faces. We found significant differences in FPVs across the three groups by a one-way repeated measures ANOVA [*F*(2,23) = 268.34, *p* < 0.001] and post-hoc Wilcoxon tests [*ps* < 0.001].

**Fig 2:**
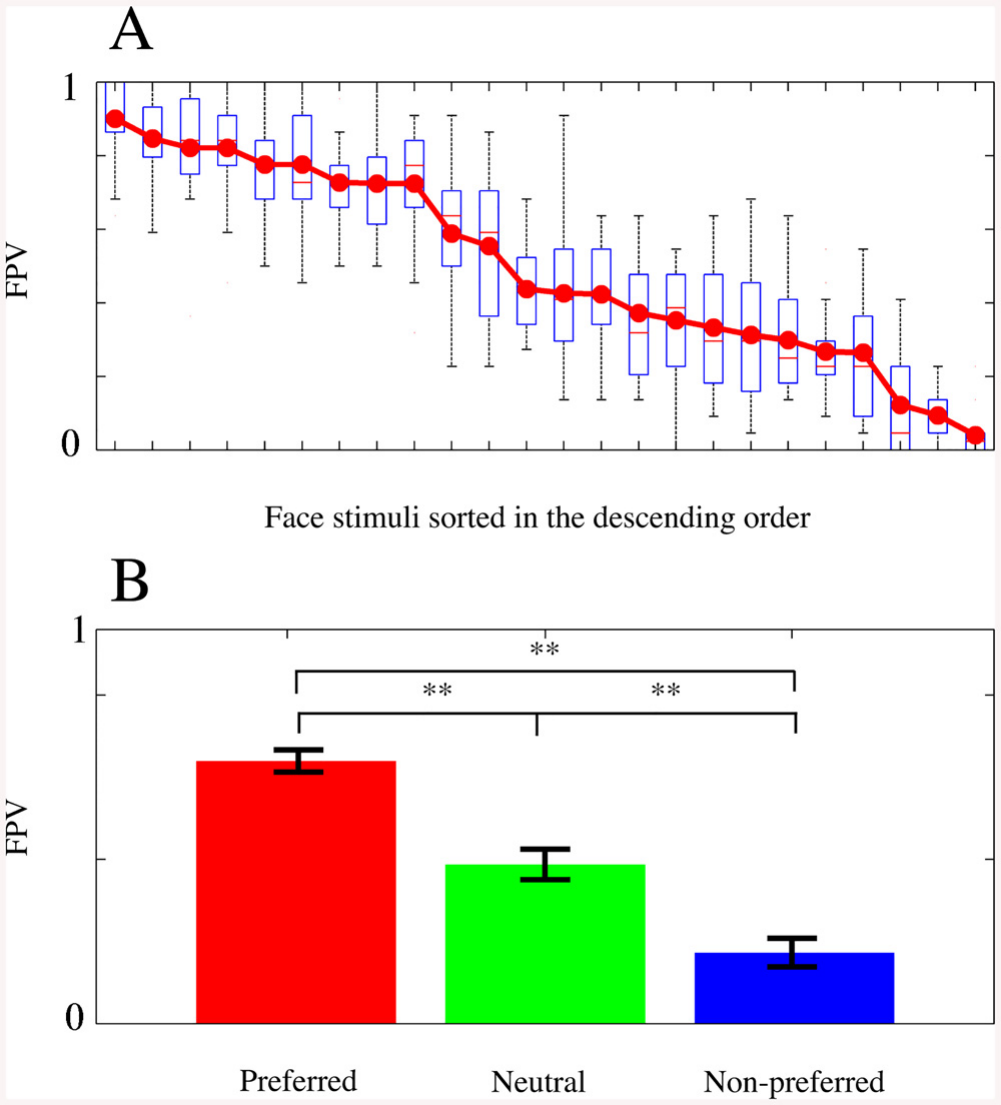
Facial preference values (FPVs) and three facial groups. (A) FPVs of all face stimuli sorted in the order of ranking. (B) Three facial groups according to the ranked FPVs and their statistical differences by paired *t*-tests (double asterisk: *p* < 0.01). The error bars indicate the standard deviations in each group.

The statistical analyses above revealed that there were no effects of gender and order of the face stimulus on the participants’ selection. Therefore, we could further analyze FPVs without considering gender and presentation order. Fig 2A shows the FPV of every stimulus averaged across all participants in a descending order and Fig 2B the three preference groups (preferred, neutral, and non-preferred) determined by the FPV ranking. The subsequent EEG analysis with face preference was performed with the FPVs from all participants.

### Modulation of EEG oscillatory powers with facial preference

#### Passive viewing task

**Fig 3:**
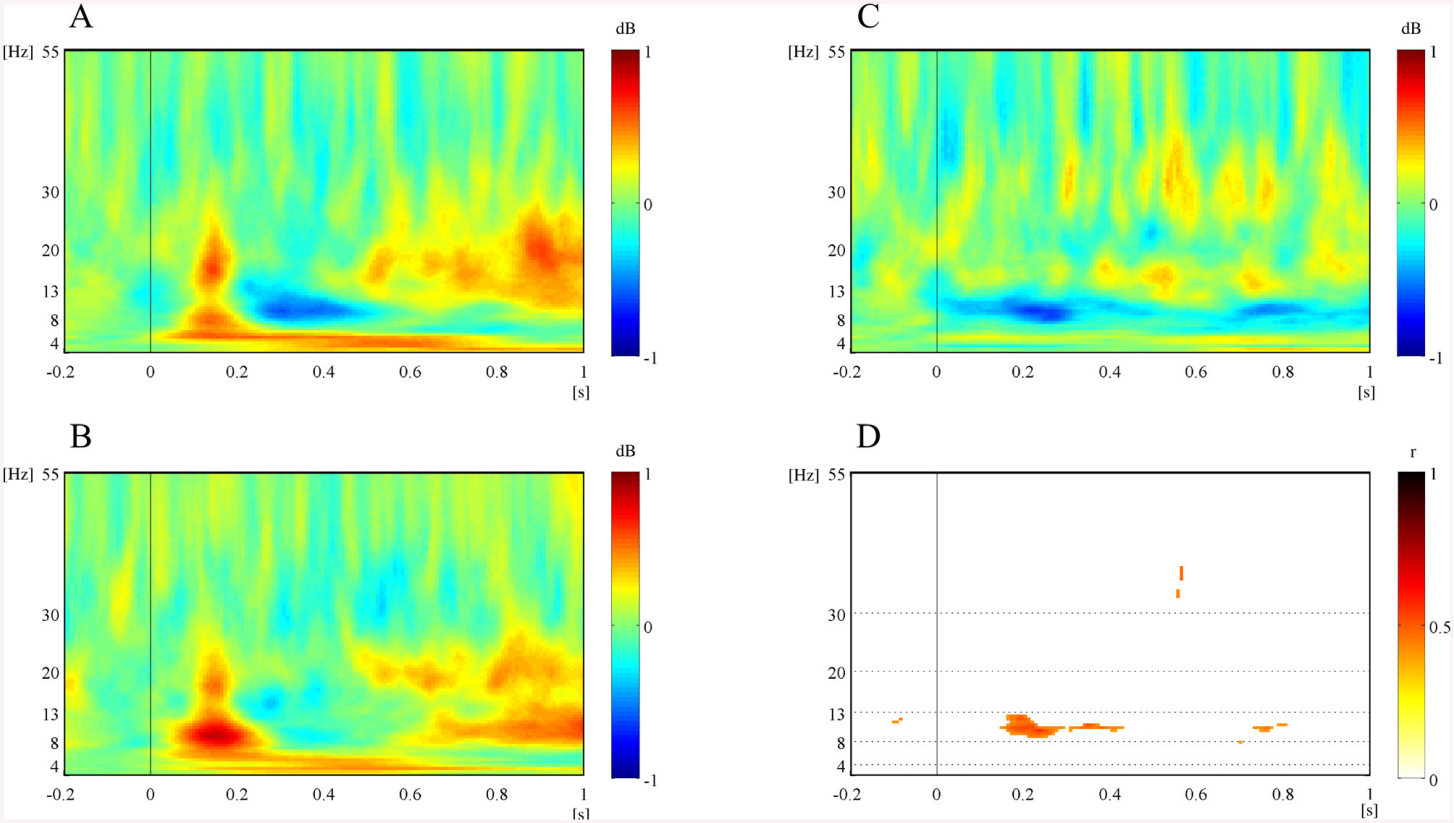
Grand average power spectrograms over the left fronto-central regions (FC3, FC1, C1, C3) in the passive viewing task. (A) Power spectrogram to the preferred face group. (B) Power spectrogram to the non-preferred face group. (C) Power differences between the preferred and non-preferred face groups. (D) Linear correlation coefficients between all individual oscillatory powers and FPVs across all face stimuli.

**Fig 4:**
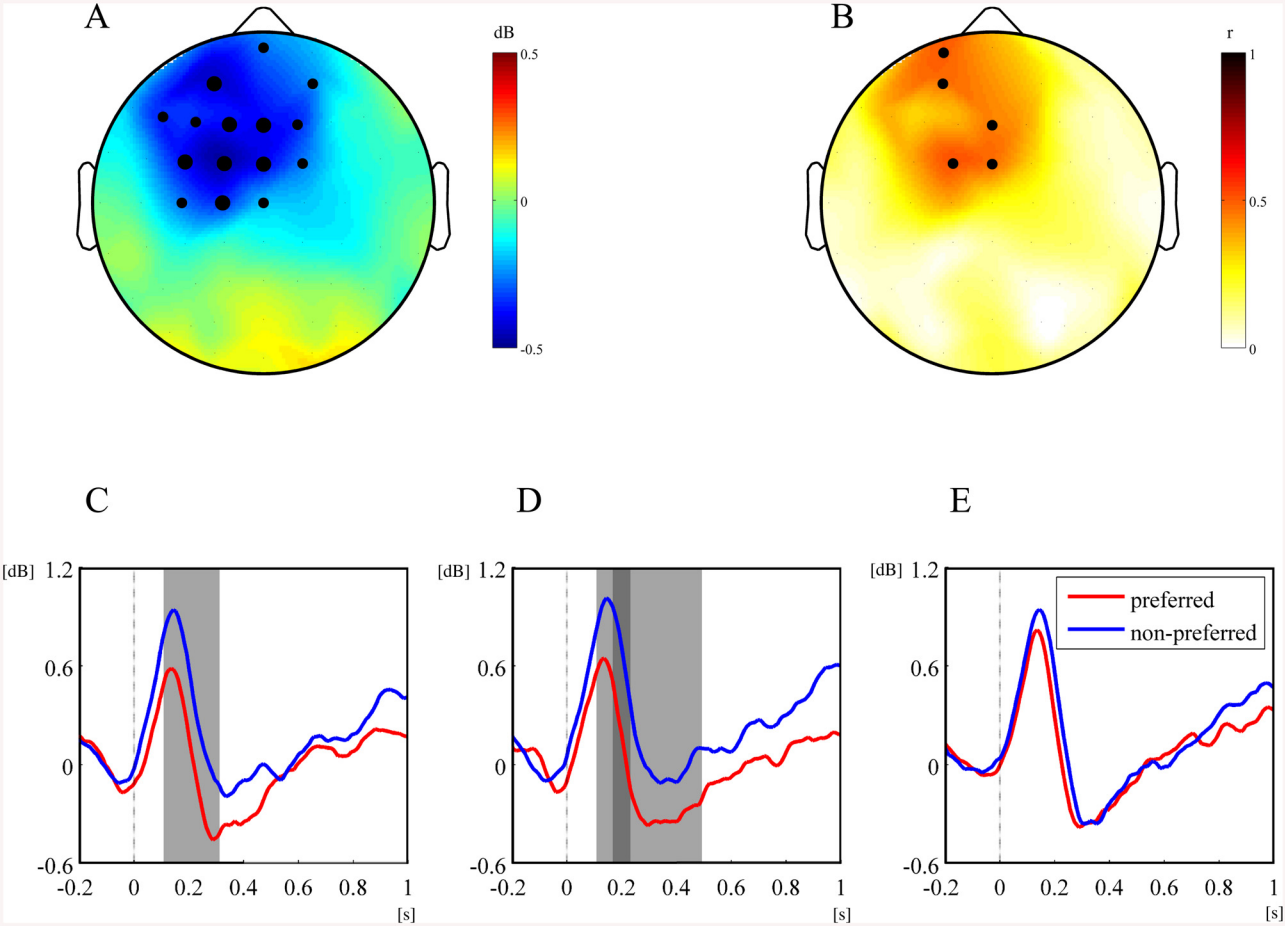
Statistical differences in alpha power between the preferred and non-preferred face groups in the passive viewing task. (A) The topographical differences in alpha power between the preferred and non-preferred face groups for 0.15 and 0.4 s. The size of the black circles indicates statistical significance level (small circle: *p* < 0.05; large circle: *p* < 0.01) by paired *t*-tests. (B) Linear correlation coefficients between alpha power and FPVs for 0.15 and 0.4 s. The size of the black circles indicates statistical significance level (small circle: *p* < 0.05; large circle: *p* < 0.01) by linear correlation analysis. (C–E) Two time courses of average alpha powers corresponding to the preferred and non-preferred face groups and the significant differences between them at the left frontal (F3, C), mid frontal (Fz, D), and right frontal (F4, E) sites (paired *t*-tests; light gray: *p* < 0.05, dark gray: *p* < 0.01).

To represent overall time-frequency patterns of the EEG oscillatory power related to facial preference, we constructed the spectrograms of the average EEG oscillatory power corresponding to the preferred and non-preferred face groups as well as the spectrograms of the average linear correlation coefficients between EEG oscillatory powers and the FPVs. Fig 3 shows the average spectrogram across the left fronto-central regions (FC3, FC1, C1, C3) during the passive viewing task. As shown in Fig 3, the preferred face group produced significantly less alpha power than the non-preferred group did in the period between 0.15 and 0.4 s after stimulus onset. The linear regression analysis between alpha powers and FPVs across all participants revealed that alpha event-related desynchronization (ERD) was significantly correlated with FPVs (0.5 < *r* < 0.6). Such significant differences in oscillatory powers between the preferred and non-preferred groups were predominantly observed in the alpha band (8–13 Hz) but not in other bands. Therefore, we focused on the alpha power for the period of significant differences (0.15–0.4 s) to perform further paired *t*-tests and linear regression analyses as shown in Fig 4. The topographical map of alpha power in the period of 0.15 to 0.4 s post-stimulus showed that the preferred group induced larger increases in alpha ERD at the left fronto-central regions.

To further examine the lateralization of alpha ERD in the frontal regions, we compared the time course of alpha powers between the left (F3, Fig 4C), mid (Fz, Fig 4D), and right (F4, Fig 4E) frontal sites for the preferred and non-preferred groups and conducted paired *t*-tests for each site. The results showed that increased alpha ERD for the preferred group occurred for 0.15–0.3 s at the left frontal region and for 0.1–0.5 s at the mid frontal region, compared with the non-preferred group. A significant difference in alpha power between the preferred and non-preferred groups was not observed at the right frontal region. These results demonstrate the spectral characteristics of EEG; by passively viewing the faces, which would be preferably selected later, increased alpha ERD was measured during the earlier period (0.15–0.4 s) with a frontal asymmetry showing more enhanced left frontal regions than right regions (S2 File).

#### Active choice task

**Fig 5:**
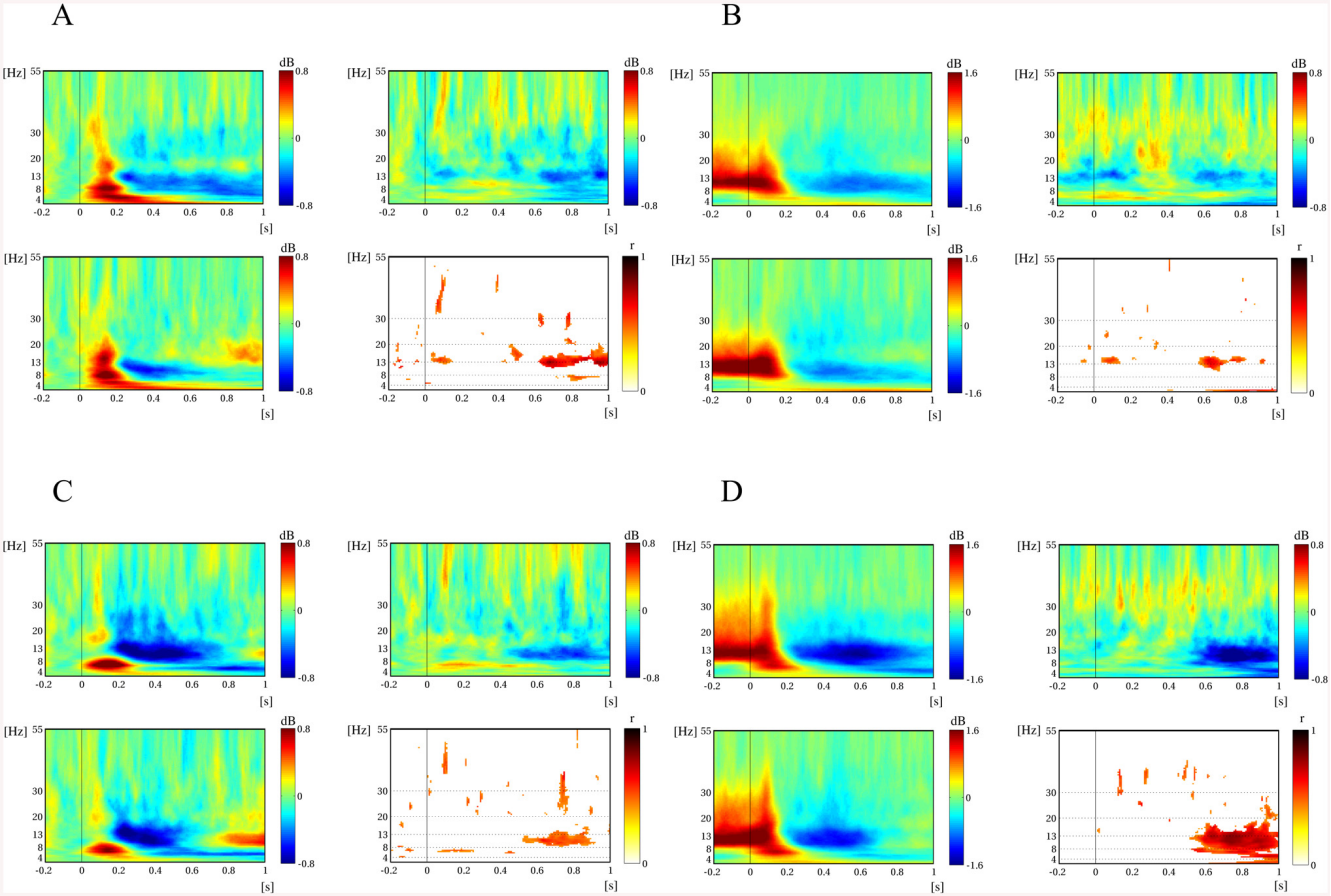
Representative spectrograms for power and linear correlation coefficients during the active choice task. (A) Fronto-central (FCz) region in the first epoch. (B) Fronto-central (FCz) region in the second epoch. (C) Parieto-occipital (POz) region in the first epoch. (D) Parieto-occipital (POz) region in the second epoch. Each panel shows the power spectrogram for the preferred (left top) and non-preferred (left bottom) face groups, the power differences between them (right top) and linear correlation coefficients between spectral power and FPVs across all face stimuli (right bottom).

We analyzed the EEG data during the first and second stimulus presentation in the active choice task in a similar way to the passive viewing task. Fig 5 illustrates the four representative spectrograms of EEG oscillatory powers and linear correlation coefficients over the frontal-central (FCz) and parieto-occipital (POz) regions. Similar to the passive viewing task, differences in oscillatory power between the preferred and non-preferred face groups were dominantly observed in the alpha band for both first and second stimulus presentation epochs of the active choice task (Fig 5). However, the time period when these differences were observed differed from the passive viewing task. Differences were mainly observed between 0.6 and 0.85 s after stimulus onset in both the first and second epochs of the active choice task. From the perspective of spatial distribution, the significant differences in alpha ERD between the preferred and non-preferred groups were distributed across all brain regions in the first epoch, whereas they were distributed largely over the posterior rather than anterior regions in the second epoch. Overall, larger differences between preferred and non-preferred groups were observed in the active choice task than in the passive viewing task.

**Fig 6:**
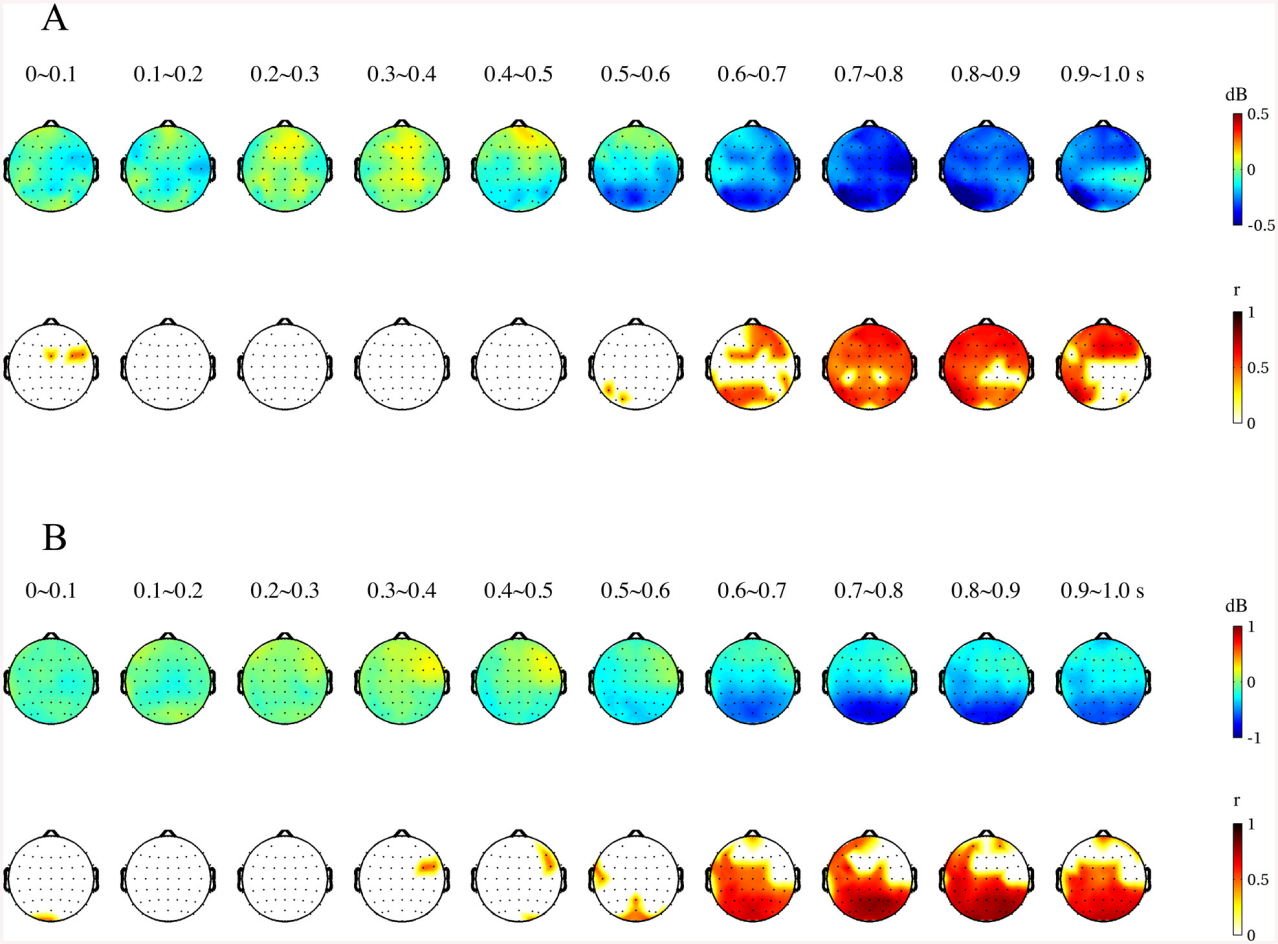
Topographical differences in alpha power between the preferred and non-preferred face groups (top) and linear correlation coefficients between alpha power and FPVs across all face stimuli (bottom); (A) in the first epoch and (B) in the second epoch during the active choice task.

**Fig 7:**
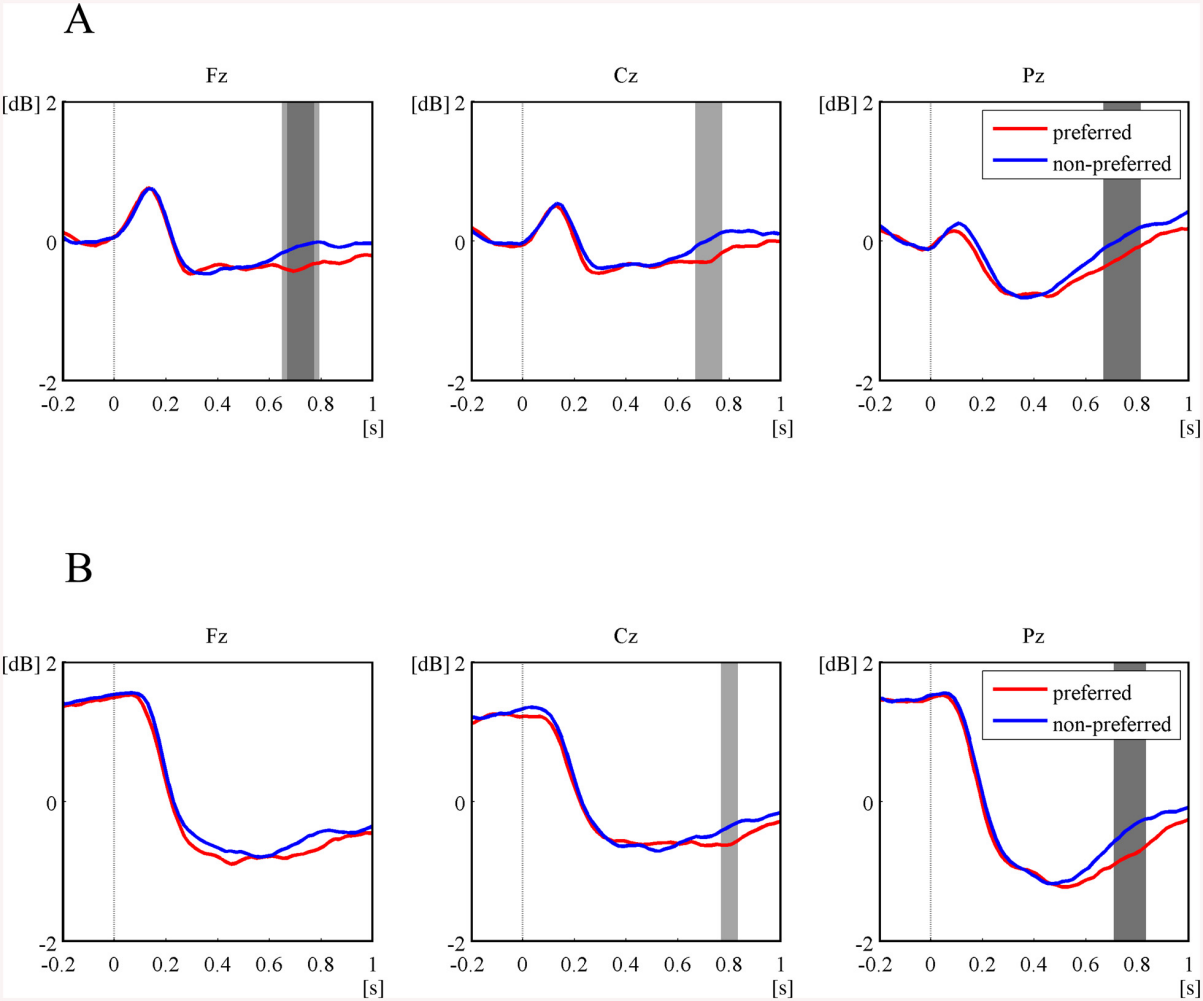
Two time courses of average alpha powers across the preferred and non-preferred face groups and significant differences between them at the mid frontal (Fz, left), central (Cz, mid), and parietal (Pz, right) sites; (A) in the first epoch and (B) in the second epoch during the active choice task (paired *t*-tests; light gray: *p* < 0.05, dark gray: *p* < 0.01)

For the examination of the spatial and temporal changes in alpha power, we constructed topographical maps of the difference in alpha power between the preferred and non-preferred groups as well as the linear correlation coefficients between FPVs and alpha ERD during the course of the active choice task (Fig 6). Each topographic map represents power difference or correlations for a 0.1-s non-overlapping sliding window. From the topographical maps, we observed that the preferred face group induced larger alpha ERD 0.5 s post-stimulus in the first epoch of the active choice task. The larger alpha ERD by the preferred group was observed over the whole brain. In addition, significant linear correlations between alpha ERD and FPV were observed approximately 0.6 s post-stimulus and over the whole brain. In contrast, during the second epoch of the active choice task, the spatial distributions of both differences in alpha power and linear correlation coefficients were partially observed over the posterior regions (S2 File). In further analysis, we constructed the three representative time courses of alpha power at Fz, Cz, and Pz responding to the preferred and non-preferred groups in the first and second epochs (Fig 7). Two-tailed paired *t*-test analyses throughout all time periods revealed larger alpha ERD for the preferred group at Fz, Cz, and Pz between 0.65 and 0.8 s post-stimulus in the first epoch but only at Pz and Cz in the second epoch.

**Fig 8:**
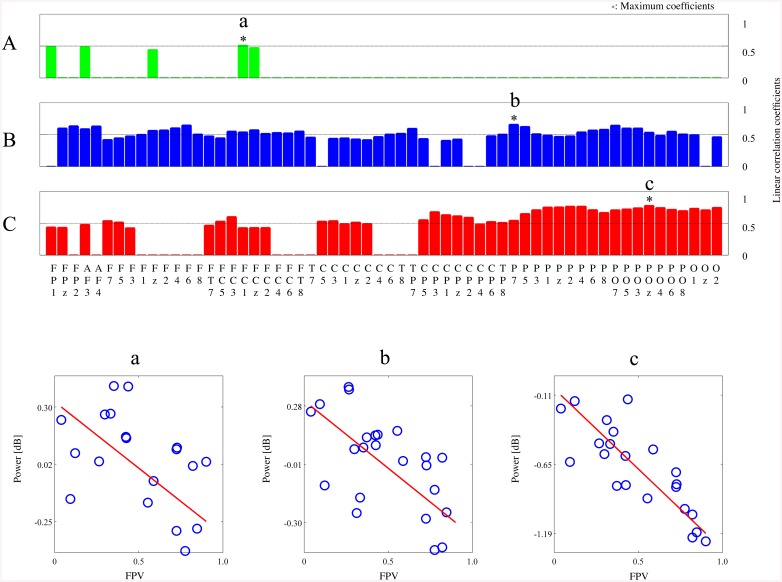
Linear correlation coefficients between alpha power and FPVs. (A) for the early period (0.15–0.4 s) in the passive viewing task, (B) for the late period (0.65–0.8 s) of the first epoch in the active choice task, and (C) for the late period (0.65–0.8 s) of the second epoch in the active choice task. The three bottom panels show the relationship between alpha powers and FPVs across all face stimuli at the maximum correlation coefficients of the passive viewing task (a), of the first epoch (b), and of the second epoch (c) in the active choice task.

We further investigated how the linear correlation coefficients changed between different tasks. Based on the statistical analysis results above, we determined the specific time periods when maximum differences in alpha power between the preferred and non-preferred groups were observed; these consisted of 0.15–0.4 s in the passive viewing task and 0.65–0.8 s for both epochs of the active choice task. We averaged the alpha power values within each of these periods and correlated them with FPV. Fig 8 depicts three sets of linear correlation coefficients for all sites with a satisfactory significance level (*p* < 0.05) and three representative plots showing the maximum correlations between alpha power and FPV for each period. In the passive viewing task, 5 out of 60 sites exhibited significant linear correlation coefficients. The coefficients varied from 0.42 to 0.50 with the maximum of 0.50 observed at FC1. All the significant correlations were observed only at the anterior sites. In the first epoch of the active choice task, 54 out of 60 sites showed significant linear correlations across all brain regions. The coefficients varied from 0.41 and 0.66 with the maximum of 0.66 observed at P7. In the second epoch of the active choice task, 44 out of 60 sites exhibited significant correlations, mostly biased over the posterior regions. The coefficients varied from 0.42 and 0.78 with the maximum of 0.78 observed at POz. These results showed a trend that linear correlations between alpha power and FPV tended to increase from the passive viewing task to the second epoch of the active choice task, with the maximally correlated sites changing from FC1 in the passive viewing to P7 and POz in the active choice tasks.

## Discussion

In the present study, we identified induced EEG oscillatory power during passively viewing or actively selecting preferred face images. Our main findings can be summarized as follows: 1) Among the various oscillatory rhythms, alpha oscillation ranging between 8 and 13 Hz was sensitive to facial preference, and the alpha oscillatory power for the preferred faces was more decreased than that for the non-preferred faces in both the passive viewing and active choice tasks. 2) During the passive viewing task, significant differences in alpha power between the preferred and non-preferred face groups were observed at a relatively early period (0.15–0.4 s after stimulus onset) in the left frontal region. By contrast, these differences were markedly observed during the late period (0.65–0.8 s) across the whole brain (first epoch) or at the posterior region (second epoch) during the active choice task. 3) The induced alpha power related to facial preference was linearly correlated with the FPV, showing stronger correlations during the active choice task.

### Alpha activity reflects top-down processes dependent on cognitive resources

Cognitive interpretations of alpha activity still remain controversial. From the classical perspective, alpha activity has been regarded as a neural representation of cortical idling [39]. However, another hypothesis proposes that alpha activity serves as inhibitory gating in various cognitive processes in which alpha activity actively inhibits task-irrelevant cortical areas [40,41]. These different hypotheses were mainly attributed to the peculiar characteristics of alpha activity. While other frequency bands showed only task-relevant event-related synchronization (ERS) related to specific cognitive processes, alpha rhythms inversely reflected both task-relevant ERD and task-irrelevant ERS [42].

The current study supports he hypothesis that alpha activity is closely related to top-down processes that need more cognitive resources such as attention [41,43,44] and working and long-term memory [31,45,46] rather than automatic bottom-up processes. Specifically, Stein *et al*. demonstrated that alpha rhythm is an extreme example of top-down processing by showing the crucial role of mid-frequency (4–12 Hz) interaction during information processing of visual stimuli across hierarchical structures of cat visual cortex during a go/no-go task [47]. The engagement of alpha activity in top-down processes has also been reported in the field of facial information processing. By probing the effect of semantic knowledge (famous vs. non-famous) on induced EEG oscillations during episodic memory task (old/new paradigm), Zion-Golumbic *et al*. reported that the posterior alpha ERD was larger for famous faces than for non-famous faces during memory encoding and also larger for old faces than for new faces during memory retrieval. With respect to these results, the authors proposed that alpha ERD was increased by the cognitive demand of semantic knowledge, and this alpha ERD characteristic reflected the cortical activation related to mental ability such as long-term memory [29].

In line with these findings, our result that alpha activity was modulated by facial preference demonstrates the characteristics of alpha activity related to the top-down process of subjective facial preference, which demands more cognitive resources for emotional perception, visual attention, remembering, retrieval of the encountered faces, and contextual comparison for facial preference judgment of consecutively presented faces.

### Preferred faces need greater attentional resources

Facial information processing needs cognitive resources [4,48–50]. More attractive faces capture more cognitive (attentional) resources accompanying larger brain responses [50,51]. Several studies have demonstrated that top-down processes related to facial beauty and attractiveness allocate more cognitive resources rather than bottom-up processes triggered by sexual attractiveness [48,49]. Recently, Girges *et al*. reported that an increase in alpha ERD at the parieto-occipital regions reflected activity of the face-specific posterior STS for higher attentional efforts allocated to perceiving upright facial motion [32]. Consistent with these findings, our results also demonstrated that alpha power for the preferred face group was more decreased than for the non-preferred face group, because the preferred faces required more intensive information processing that was likely to be reflected by alpha ERD.

### Motivational significance of the presented face makes a difference in the left frontal alpha ERD

In the passive viewing task, significant differences in alpha power between the preferred and non-preferred faces were found in the early period (0.15–0.4 s) with an apparent frontal lateralization, showing an increase in alpha ERD for the preferred faces at the left frontal region. This result is consistent with the frontal EEG asymmetry as a mediator of emotion [52]. One of the well-known hypotheses in emotional and facial processing, the valence-specific hypothesis [53], posits that positive emotional or social approach stimuli are highly associated with neural activation of the left frontal regions (increase in alpha ERD), while negative or withdrawal stimuli are related to neural activation in the right frontal regions [54,55]. Contrary to the active choice task, there was no need of either judging facial preference or remembering the presented faces in the passive task. Therefore, we posit that the differences in alpha ERD between the preferred and non-preferred face groups and the left frontal asymmetry can be attributed to the motivational approach or withdrawal derived from the preference of an encountered face.

### Formation of facial preference reflected on the alpha ERD in the posterior region

The linear relationship between facial preference and alpha power was strongly found at the posterior regions in the second epoch of the active choice task. Several studies demonstrated that posterior regions play a crucial role in memory and social interactions, showing modulation of alpha activity as follows. By using human face stimuli with two factors, facial identity and orientation, Jokisch and Jenson showed that posterior alpha activity was increased for the working memory retention of face identities in the ventral stream, and occipital alpha activity was decreased for the retention of face orientation in the dorsal stream. These results support the association of alpha activity with a general suppression mechanism by providing evidence that increased posterior alpha power may reflect the facilitation of engagement of the ventral stream through the inhibition of the dorsal stream during facial information processing with working memory [56]. Breton *et al*. reported that posterior alpha ERD was found in cognitive processes related to facial hierarchy. In this study, participants were asked to view high-, middle-, or low-ranking faces in comparison with themselves where the ranking was determined by a simple visual perception task. When they encountered high-ranking faces, an enhanced alpha ERD at posterior regions was observed [57]. The parietal cortex is implicated not only in physical distance [58–60] but also in social distance [33,61–63]. As a part of the social brain network, the posterior parietal cortex plays a critical role in the judgment of social distance to external objects by self-referential operations that transform the physical and spatial information of external objects into egocentric coordinates for action [34]. Specifically, Giardina *et al*. confirmed that the posterior parietal cortex plays an important role in the processing of spatial and social distance that could be manipulated by means of face or object stimuli [33]. Our results obtained during the second epoch of the active choice task are in line with these previous findings regarding the role of posterior regions in social distance. Therefore, it is likely that the assessment of social distance related to formation of preference was strongly reflected as alpha ERD in the posterior region.

### Conclusion and future works

In this study, we found that the formation of subjective facial preference strongly reflected on the EEG alpha oscillatory power. Based on these findings from the two tasks, we suggest that the spatial and temporal changes on EEG alpha power provide neural evidence of subjective facial preference and show the potential ability of EEG to predict subjective preferences for human faces. Our future research will focus on the development of a reliable prediction model for subjective preferences for human faces and will extend into the neuromarketing field by means of EEG signal processing techniques.

## Supporting Information

S1 File Facial preference values. (XLSX)

S2 File All relevant alpha powers for both passive viewing and active choice tasks. (ZIP)
